# Negativity vs. purity and entropy in witnessing entanglement

**DOI:** 10.1038/s41598-023-31273-9

**Published:** 2023-03-21

**Authors:** James Schneeloch, Christopher C. Tison, H Shelton Jacinto, Paul M. Alsing

**Affiliations:** Air Force Research Laboratory, Information Directorate, Rome, NY 13441 USA

**Keywords:** Quantum information, Quantum metrology

## Abstract

In this paper, we explore the value of measures of mixedness in witnessing entanglement. While all measures of mixedness may be used to witness entanglement, we show that all such entangled states must have a negative partial transpose (NPT). Where the experimental resources needed to determine this negativity scale poorly at high dimension, we compare different measures of mixedness over both Haar-uniform and uniform-purity ensembles of joint quantum states at varying dimension to gauge their relative success at witnessing entanglement. In doing so, we find that comparing joint and marginal purities is overwhelmingly (albeit not exclusively) more successful at identifying entanglement than comparing joint and marginal von Neumann entropies, in spite of requiring fewer resources. We conclude by showing how our results impact the fundamental relationship between correlation and entanglement and related witnesses.

## Introduction

Quantum entanglement is the principal resource consumed in many applications of quantum information such as quantum computing^1–3^, communication^4–9^, and enhanced metrology^10–12^. Understanding its fundamental nature goes hand-in-hand with developing adequate techniques to fully characterize it in the exceptionally high-dimensional systems being employed today, such as: quantum computation on 127-qubit states^13^, boson samplers in $10^{30}$-dimensional state spaces^14^ or in pairs of particles entangled in high-dimensional degrees of freedom^15^. With such high-dimensional systems requiring analysis, it is important to investigate the tools we use to characterize entanglement in these systems, and find a suitable standard to gauge their relative sensitivity and scalability.

In this article, we begin in “Foundation: entanglement from mixedness and majorization” by illustrating how all measures of mixedness can be used to witness entanglement. Then, in “Result: the negativity encompasses mixedness-based entanglement witnesses” we provide a short proof verifying that all states whose entanglement can be witnessed via mixedness measures must also have a negative partial transpose (NPT), where this negativity is an entanglement witness^16^ in its own right.

Following our proof of the relative strength of the negativity, we consider in “Results: quantum state purity vs von Neumann entropy in entanglement witnessing” the case when the negativity is intractable to determine experimentally, but various measures of mixedness are easier to obtain. Toward that end, we use Monte-Carlo sampling of large uniform ensembles of states from low to high dimension (i.e., from two-qubit to two-quDit for $\text {D}=10$) to compare how well two popular measures of mixedness demonstrate entanglement with respect to one another. In this section, we compare the effectiveness of witnessing entanglement through comparisons of joint and marginal von Neumann entropy ($S_{1}({\hat{\rho }}_{AB})$ and $S_{1}({\hat{\rho }}_{A})$, respectively, where subscript 1 represents the Renyi entropy of order $\alpha =1$) to comparisons of the joint and marginal purity given as $\text {Tr}[{\hat{\rho }}_{AB}^{2}]$ and $\text {Tr}[{\hat{\rho }}_{A}^{2}]$, respectively. To facilitate a simpler side-by-side comparison, we use the negative logarithm of the purity given as the quantum collision entropy $S_{2}({\hat{\rho }})$ instead of the purity itself. From our data, we find from a uniform ensemble of states, that comparing purities is overwhelmingly more successful at witnessing entanglement than comparing von Neumann entropies, despite requiring fewer resources to obtain.

Even with the overwhelming advantage demonstrated in the uniform ensemble of states that comparing purities has over comparing von Neumann entropies, we were also able to uncover a rare class of anomalous states shown in “Vanishing likelihood of anomalous states in uniform purity ensemble” for which either the purity or the von Neumann entropy can be made advantageous. Besides this, we were able to prove in “Relative effectiveness for different classes of states” that for all states with maximally mixed marginals, and for all pure states, comparing purities is the more advantageous entanglement witnessing strategy. To accomplish this, we used analytic upper and lower bounds to von Neumann entropy at constant purity (discussed in Appendix D of the Supplementary Material).

In addition to comparing joint vs marginal $S_{1}$ and $S_{2}$ at witnessing entanglement, we also consider in “Increased sensitivity when using higher-order entropies” how the sensitivity in the entanglement witness appears to grow with the Renyi order $\alpha$, both analytically for Werner states, and numerically with our random samples. We next explore in Discussion: correlations vs negativity in witnessing entanglement the relationship between the negativity of the partial transpose and the relationship between correlation and entanglement. Where many correlation-based entanglement witnesses do so by effectively verifying a negative conditional entropy, the negativity may be seen to supersede many of these inequalities, but not all of them.

Note that here and throughout the paper we distinguish correlation from entanglement in the following way. We use correlation as a statistical relationship between measurable observables that enhances predictability (e.g., ${\hat{Q}}_{A}$ and ${\hat{Q}}_{B}$ of joint system *AB* are correlated if knowing the outcome of ${\hat{Q}}_{A}$ improves one’s ability to predict the outcome of ${\hat{Q}}_{B}$). By contrast, entanglement is defined solely as the lack of a joint quantum state (e.g., of *AB*) to factor out as a product of individual quantum states for each subsystem (or for mixed states, a classical mixture thereof). While it can be argued (particularly for pure states) that entanglement implies the presence of correlations, this is tangential to its primary definition from nonseparability. We make this distinction because there are entanglement witnesses that do not provide any direct information about the correlations present within a system (see for example, the negative partial transpose criterion discussed in the next section).

## Foundation: entanglement from mixedness and majorization

In classical probability, joint distributions are never less mixed than the marginal distributions obtained from them. In the language of Shannon entropy, the joint entropy is never less than the marginal entropy; two random variables never take less information to communicate than one. However, this need not be the case when comparing the mixedness of joint and marginal quantum states.

To quantify the mixedness of quantum states, we measure the mixedness of the probability distribution generated by the eigenvalues of the density matrix. Given a probability distribution of *N* outcomes $\{p_{i}\}_{i=1}^{N}$, we define the probability vector $\vec {p}$ as the *N*-dimensional vector whose components are the probabilities $\{p_{i}\}_{i=1}^{N}$. In addition, we provide the following definition for an arbitrary measure of mixedness:

### Definition 1

A measure of mixedness is any continuous Schur-concave^17^ function ${\mathscr {F}}$ of a probability vector $\vec {p}$ with minimum value zero for “pure” probability distributions (in which one outcome contains all probability). The measure of mixedness for a quantum density matrix is of the probability vector of its eigenvalues.

Note that a Schur-concave function is a function $f:{\textbf{R}}^{n}\rightarrow {\textbf{R}}$ such that for any pair of vectors $\vec {u}$ and $\vec {v}$ in ${\textbf{R}}^{n}$ in which $\vec {u}$ majorizes $\vec {v}$, it must follow that $f(\vec {u})\le f(\vec {v})$. For two probability vectors $\vec {p}$ and $\vec {q}$ in ${\textbf{R}}^{n}$, we denote the statement $\vec {p}$ majorizes $\vec {q}$ as $\vec {p}\succ \vec {q}$. In short, $\vec {p}\succ \vec {q}$ if for all *k* from 1 to the dimension of $\vec {p}$, the sum of the *k* largest elements of $\vec {p}$ is greater than or equal to the corresponding sum of the *k* largest elements of $\vec {q}$.

Such measures of mixedness ${\mathscr {F}}$ are also maximum for the uniform distribution, and monotonically increase under any mixing operations that replace elements of $\vec {p}$ with ones closer to the average value of the elements chosen. In particular, the value of ${\mathscr {F}}$ must increase for any distribution where pairs of unequal elements are re-distributed to bring them closer to their arithmetic mean (known as Robin-Hood operations). All forms of entropy, including the von Neumann, Renyi, and Tsallis entropies are Schur-concave, and serve as measures of mixedness.

In examining measures of mixedness, there is a disconnect between showing that one distribution $\vec {q}$ is obtainable from another $\vec {p}$ through mixing operations, and that ${\mathscr {F}}(\vec {q})>{\mathscr {F}}(\vec {p})$ for some measure of mixedness. When (and only when) $\vec {q}$ can be obtained through a sequence of Robin-Hood mixing operations on $\vec {p}$, we say that $\vec {p}$ *majorizes* $\vec {q}$, denoted by $\vec {p}\succ \vec {q}$. Alternatively, when the probability eigenvalues of density matrix ${\hat{\rho }}$ majorize the probability eigenvalues of density matrix ${\hat{\sigma }}$, then we say that ${\hat{\rho }}$ majorizes ${\hat{\sigma }}$ or that ${\hat{\rho }}\succ {\hat{\sigma }}$. If $\vec {p}\succ \vec {q}$, then we know that the distribution $\vec {p}$ is more pure (less mixed) than $\vec {q}$ because there exists a series of mixing operations to obtain $\vec {q}$ from $\vec {p}$. That said, there are pairs of probability distributions where neither majorizes the other (here called incomparable), even though mixedness measures ${\mathscr {F}}$ are well-defined for both. This is because, for such incomparable probability distributions represented by $\vec {p}$ and $\vec {q}$, one measure of mixedness ${\mathscr {F}}$ might show that $\vec {p}$ is more mixed than $\vec {q}$ via ${\mathscr {F}}(\vec {p})>{\mathscr {F}}(\vec {q})$, while another measure ${\mathscr {G}}$ might show that $\vec {p}$ is less mixed than $\vec {q}$ by ${\mathscr {G}}(\vec {p})<{\mathscr {G}}(\vec {q})$. However, when $\vec {p}$ and $\vec {q}$ are comparable (i.e., $\vec {p}\succ \vec {q}$ or $\vec {p}\prec \vec {q}$) then all measures of mixedness will agree on whether $\vec {p}$ is less mixed than $\vec {q}$.

Unlike classical probability distributions, quantum states are special because it is possible for the joint state of two parties *AB* (given by the density matrix ${\hat{\rho }}_{AB}$) to be less mixed than the marginal state of either *A* or *B* (given by ${\hat{\rho }}_{A}$ and ${\hat{\rho }}_{B}$, respectively). For example, *AB* can be in a pure quantum state $|\psi \rangle _{AB}$, such as a Bell state, while the reduced states of *A* and *B* are both maximally mixed. This can only happen, however, if the joint state is entangled^18^. In fact, it was proven in Ref.^18^ that when ${\hat{\rho }}_{AB}$ is separable so that it has the form:

1 $$\begin{aligned} {\hat{\rho }}_{AB}^{(sep)}\equiv \sum _{i} p_{i}({\hat{\rho }}_{Ai}\otimes {\hat{\rho }}_{Bi}), \end{aligned}$$

then ${\hat{\rho }}_{AB}$ cannot be less mixed than either ${\hat{\rho }}_{A}$ or ${\hat{\rho }}_{B}$ because the probability eigenvalues of ${\hat{\rho }}_{AB}$ are *majorized* by those of both ${\hat{\rho }}_{A}$ and ${\hat{\rho }}_{B}$. This is known as the majorization criterion of separability. Since all measures of mixedness cannot decrease under majorization, the majorization criterion of separability implies: for all measures of mixedness ${\mathscr {F}}$, separable states must satisfy the relation:

2 $$\begin{aligned} {\mathscr {F}}({\hat{\rho }}_{AB}^{(sep)})\ge \max \{{\mathscr {F}}({\hat{\rho }}_{A}),{\mathscr {F}}({\hat{\rho }}_{B})\}, \end{aligned}$$

here called the mixedness criterion to distinguish it from majorization. However, the converse statement that all states satisfying the mixedness criterion satisfy the majorization criterion is demonstrably false. If a state satisfying the mixedness criterion for one measure of mixedness implied that the majorization criterion was satisfied, then it would also imply that the mixedness criterion is satisfied for all measures of mixedness. This is false because there exist states whose entanglement may be witnessed with one measure of mixedness, but not with another.

Comparing joint and marginal mixedness forms the basis of a broad class of entanglement witnesses. In addition to these entanglement criteria, there is another historical criterion relying on the form of the density matrix for separable states. Twenty-seven years ago, Peres^16^ showed that where separable states (1) factor into products of states for each particle, and where the transpose of a density matrix is another valid density matrix, the *partial* transpose of a separable state must also be a valid density matrix. Any state whose partial transpose yields a matrix with negative eigenvalues cannot be separable, and is therefore entangled. These entangled states are known as Negative-Partial-Transpose or NPT for short. Not all entangled states are NPT (though all 2-qubit entangled systems are^19^), but it is a simple criterion to calculate from the density matrix, and functions based on these partial-transpose eigenvalues have been used as entanglement monotones (e.g., the negativity ${\mathscr {N}}({\hat{\rho }})$ and log-negativity $E_{{\mathscr {N}}}({\hat{\rho }})$).

While measures of mixedness are well-defined functions over all density matrices, it is possible (and common) for two density matrices to be incomparable with respect to each other (i.e., where neither density matrix majorizes the other). This incomparability suggests that there are states whose entanglement cannot be witnessed by comparing one measure of mixedness, but can by another, which motivates this study. Beyond this, we can also compare the set of states witnessed by violating the majorization criterion, to that of other separability criteria.

Before we show the details of our study comparing the relative effectiveness of different measures of mixedness at witnessing entanglement, we provide a short proof that the negativity of the partial transpose actually encompasses all comparisons of joint and marginal mixedness in their ability to witness entanglement. In particular, we prove that the set of states whose entanglement is witnessed by violating the majorization criterion (including those from violating (2)) is contained within the set of NPT states. In short, there are no entangled states violating the mixedness criterion (2) that are not also NPT, which is similarly easy to compute.

## Result: the negativity encompasses mixedness-based entanglement witnesses

### Theorem 1

Given a joint density matrix ${\hat{\rho }}_{AB}$, if the mixedness criterion (2) is violated, then ${\hat{\rho }}_{AB}$ is NPT. Equivalently, the set of NPT states contains the set of states violating the mixedness criterion (2).

### Proof

In 1998, the Horodeckis^20^ proved that all states with a positive partial transpose (PPT) are undistillable. That is, one cannot take copies of PPT states (even if they are entangled) and use local operations and classical communication (LOCC) to convert those states into fewer copies of maximally entangled states. What the Horodeckis have shown is equivalent to the contrapositive statement that all distillable states have a negative partial transpose (NPT). This is not the same thing as answering whether all NPT states are distillable, which remains an open question^21^.

In 2003, Tohya Hiroshima proved^22^ that if a joint state ${\hat{\rho }}_{AB}$ is undistillable, then it must satisfy the majorization criterion. This is equivalent to the contrapositive statement that all states that violate the majorization criterion (which includes those that violate the mixedness criterion (2)) must have distillable entanglement.

Together, these two historical results validate the following deduction:

- Since all states that violate the mixedness criterion (2), must also violate the majorization criterion,
- and all states that violate the majorization criterion must also be distillable,
- and all states that are distillable must also be NPT...

it follows that all states that violate the mixedness criterion (2) must also be NPT states, thus proving Theorem 1. $\square$

By Theorem 1, we know there are no states that can violate the mixedness criterion (2) that won’t also be NPT. The negativity will witness entanglement in at least all states whose entanglement can be witnessed by violating the mixedness criterion (2). However, that does not mean that comparing measures of mixedness is obsolete.

**Figure 1:**
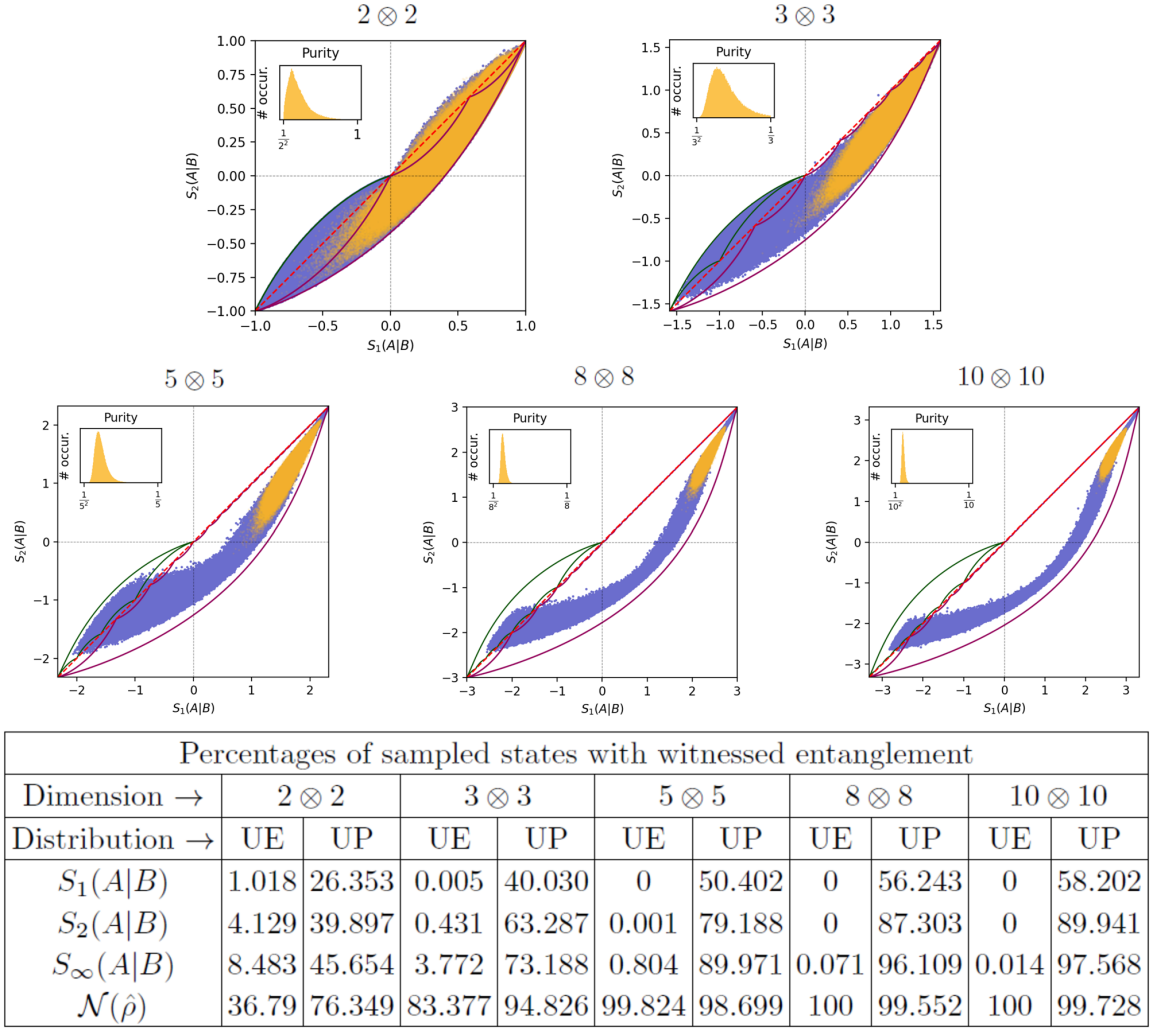
(Top) Scatterplots of $S_{2}(A|B)$ vs $S_{1}(A|B)$ and respective purity histograms for $10^{6}$ 2-quDit systems for $D=(2,3,5,8,10)$ with each plot labeled $2\otimes 2$, $3\otimes 3$, $5\otimes 5$, $8\otimes 8$,and $10\otimes 10$, respectively. The light orange scatterplots are from the fully uniform ensemble (abbreviated UE) while the blue scatterplots are from the ensemble uniform with respect to purity (abbreviated UP). The inset histograms are of the joint purity of the fully uniform ensemble). The red dotted line in each plot is where $S_{1}(A|B)=S_{2}(A|B)$. The set of all $D\otimes D$ pure states is within the green serrated blade region in the lower left quadrant (or is only a single curve for $2\otimes 2$), while the set of all $D\otimes D$ states with maximally mixed marginals corresponds to the large magenta serrated blade spanning three quadrants of the plot. The regions enclosed between the two blades also correspond to valid density matrices. (Bottom) This table gives the percentages of the total number of generated states whose entanglement was witnessed with the function in the first column.

Although the negativity of the partial transpose is a computable entanglement witness from the density matrix, the difficulty in reconstructing a density matrix from experimental data would be intractable at high dimension due to the sheer number of elements that a density matrix may contain. Although tomography is not too challenging for a state made of one or two qubits, the number of elements to be determined increases exponentially with the number of qubits. For example, any quantum state in a Hilbert space of dimension $10^{30}$ can be expressed using no more than 100 qubits. In these regimes, no attempt at full state tomography would ever be made, but entanglement can be efficiently verified by obtaining a sufficiently high fidelity between the measured state and an ideal resource state^23,24^. What we explore here are strategies for efficiently characterizing the entanglement present in high dimensional systems where a target state is not given, but the state remains too large for tomography to be performed.

When full state tomography is not feasible, it is still theoretically possible to determine the eigenvalue spectrum of an *n*-dimensional density matrix ${\hat{\rho }}$ without determining the density matrix itself. If one can determine all of the *n*-th order moments of ${\hat{\rho }}$ given as the trace $\text {Tr}[{\hat{\rho }}^{n}]$ directly from experimental data, then the eigenvalue spectrum can be obtained by solving the set of *n* eigenvalue equations. Though there exist multiple strategies for determining the second-order moment^25–27^, determining the *n*-th order moment requires measurements that interfere *n* identical copies of the quantum state, which makes determining the full eigenvalue spectrum unfeasible at high dimension as well. There also exist strategies for estimating the negativity without having to determine the density matrix. These require determining at least the third-order moment of the partial transposed density matrix, which may be obtained experimentally through random or collective measurements^28–31^. However, the *second*-order moment of the density matrix is a valid measure of mixedness in its own right, known as the purity. In what follows, we show compelling evidence that comparing the joint and marginal purity witnesses entanglement more often than comparing the joint and marginal von Neumann entropy even though the latter is more difficult to determine experimentally. Moreover, we demonstrate how states for which comparing von Neumann entropies is more successful are exceptionally rare in a uniform ensemble of density matrices.

## Results: quantum state purity vs von Neumann entropy in entanglement witnessing

In this section, we examine measures of mixedness based on the second-order moment of the density matrix (i.e., $\text {Tr}[{\hat{\rho }}^{2}]$), in comparison to the von Neumann entropy given as $-\text {Tr}[{\hat{\rho }}\log ({\hat{\rho }})]$. In particular, we show how comparing the joint and marginal state purities is almost always more successful at witnessing entanglement than comparing joint and marginal von Neumann entropies, even though fewer resources are required to determine the state purity. While the von Neumann entropy requires knowing the complete eigenvalue spectrum of the density matrix, state purities can be measured directly by interfering two identical copies of the system in an experiment^25,26^. To facilitate a side-by-side comparison of von Neumann entropy and state purity at witnessing entanglement, we consider comparing the Renyi entropies of order $\alpha$ (given by $S_{\alpha }$) without loss of generality:

3a $$\begin{aligned} S_{\alpha }(A)&=S_{\alpha }({\hat{\rho }}_{A})\equiv \frac{1}{1-\alpha }\log \Big (\text {Tr}[{\hat{\rho }}_{A}^{\alpha }]\Big ), \end{aligned}$$

3b $$\begin{aligned} \lim _{\alpha \rightarrow 1}S_{\alpha }(A)&=S_{1}(A)\equiv -\text {Tr}[{\hat{\rho }}_{A}\log ({\hat{\rho }}_{A})], \end{aligned}$$

3c $$\begin{aligned} \lim _{\alpha \rightarrow 2}S_{\alpha }(A)&=S_{2}(A)=-\log \Big (\text {Tr}[{\hat{\rho }}_{A}^{2}]\Big ), \end{aligned}$$

3d $$\begin{aligned} \lim _{\alpha \rightarrow \infty }S_{\alpha }(A)&=S_{\infty }(A)=-\log \Big (\max _{i}\{\lambda _{i}\}\Big ), \end{aligned}$$

3e $$\begin{aligned} S_{\alpha }(A|B)&=S_{\alpha }(AB)-S_{\alpha }(B). \end{aligned}$$

Here we see that $S_{1}(A)$ is the von Neumann entropy of system *A*, and $S_{2}(A)$ is a monotonically decreasing function of the purity $\text {Tr}[{\hat{\rho }}_{A}^{2}]$, known as the quantum collision entropy. In addition, $S_{\infty }(A)$ is known as the quantum min entropy, whose utility in entanglement witnessing is illustrated in “The data set of randomly generated density matrices”. We define the Renyi conditional entropy $S_{\alpha }(A|B)$ for convenience. Whenever $S_{\alpha }(A|B)$ is negative, $S_{\alpha }(AB)$ is less than $S_{\alpha }(B)$, which witnesses entanglement by violating the mixedness criterion (2). Here and throughout this paper, all logarithms are base two, since we measure entropy in bits.

### Monte Carlo simulations of random density matrices

In order to compare the effectiveness of comparing von Neumann entropies to comparing state purities as witnesses of entanglement, we performed Monte-Carlo simulations on 1 million 2-quDit systems, for $D=\{2,3,5,8,10\}$. In other words, we randomly generated these 2-quDit systems and calculated their joint and marginal von Neumann entropies and purities to see what fraction of states generated had their entanglement witnessed by each measure of mixedness. For each dimension, we generated two uniform ensembles of density matrices. The first was completely uniform over the simplex of eigenvalues (discussed in the following paragraph), while the second ensemble was uniform over the simplex for each value of purity, but with the value of purity distributed uniformly. In Fig. 1, the orange scatterplots give the fully uniform ensemble (abbreviated as UE), while the blue scatterplots give the uniform purity ensemble (abbreviated as UP). The reason for generating the second distribution is because the fully uniform ensemble of density matrices is highly non-uniform with respect to purity, producing nearly pure and nearly maximally mixed states with negligible probability at high dimension, as discussed later in this section.

#### Step 1: generating the eigenspectrum

Generating a fair sampling of random density matrices is a two-step process discussed in Refs.^32,33^. First, the eigenvalue spectrum of the density matrix is generated from a uniform distribution of probability vectors. This works because all probability vectors of dimension *N* represent valid eigenvalue spectra for density matrices of dimension *N* and vice versa. The uniform distribution of probability vectors is defined as follows. The set of probability vectors $\vec {p}$ of dimension *N* forms a hyperplane of dimension $N-1$ due to the constraint equation that the sum of all components of $\vec {p}$ add to unity. This hyperplane is further bounded into a regular $(N-1)$-dimensional simplex by the constraints that each component of $\vec {p}$ be non-negative. In Fig. 2 of the Supplementary Material, we have a diagram of the uniform distribution of eigenvalue vectors for $N=3$. As a flat surface in *N*-dimensional space, the uniform distribution of probability vectors is uniform on this surface. In Fig. 1, the orange scatterplots and histograms refer to states generated from this uniform ensemble.

**Figure 2:**
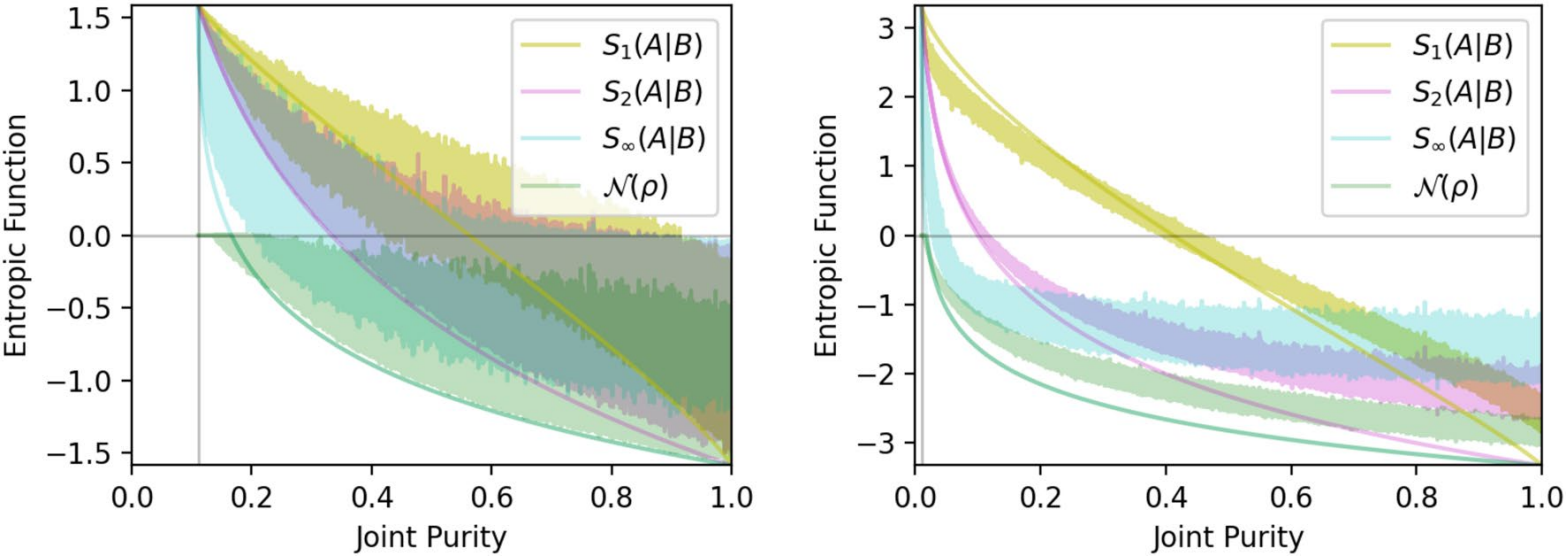
Plots showing different conditional entropy functions (and the negativity) for uniform-purity ensembles as a function of joint purity. (Left) Case of $3\otimes 3$ systems. (Right) Case of $10\otimes 10$ systems. Narrow curves of the same color plot the corresponding function of the Werner state whose purity is varied by changing the mixing parameter *p*. Note that the Werner state curves for negative the log negativity $-E_{{\mathscr {N}}}({\hat{\rho }})$ coincide with the conditional min entropy $S_{\infty }(A|B)$ where entanglement is witnessed.

It may seem that great pains are taken to generate this particular distribution of eigenvalue spectra when one could otherwise simply generate random numbers between zero and one for each eigenvalue and renormalize to set the sum equal to one. However, such a process is overwhelmingly weighted in favor of the maximally mixed state at high dimension, due to the law of large numbers (see Appendix A of Supplementary Material for details).

When studying the effectiveness of various entanglement witnesses, it is important to cover all possible values that these witnesses might take with a large enough number of randomly generated states. Pure states of two or more parties that are anything other than an uncorrelated product of component party states are entangled. Nearly-pure states with slight but sufficient correlations are also demonstrably entangled (see Eq. (8) for illustration). For increasing levels of mixedness, there are fewer entangled states consistent with that amount of mixedness^32^. However, the uniform distribution of probability vectors has only a small fraction of its total hypervolume in close proximity to a pure state. Indeed, if we take the fraction of “nearly-pure” states to be the fraction of states that have a maximum probability component of at least 1/2, then that fraction of total probability vectors that are nearly pure would be $N\times 2^{(1-N)}$, which decreases exponentially toward zero for large *N*. Alternatively, if we take the fraction of “nearly maximally mixed” states to be those with a purity ${\mathscr {P}}$ between 1/*N* and $1/(N-1)$, one can show that the fraction of nearly maximally mixed states decreases even faster at high dimension (see discussion in Appendix B of Supplementary Material for details and histograms in Fig. 1 for examples).

In order to better cover the full range of values that the quantum entropy can take, we created a second ensemble of probability vectors which, for a fixed value of purity, is otherwise uniform on the probability simplex. If we constrain the *N*-dimensional probability vectors on the simplex to also have a constant purity ${\mathscr {P}}$, the result is the intersection of a sphere of radius $\sqrt{{\mathscr {P}}-1/N}$ centered at the maximally mixed state (as illustrated in Fig. 2 of the Supplementary Material) intersecting the simplex. These uniform spherical slices of the probability simplex are far from straightforward to generate, but are described in Ref.^34^. With these uniform spherical slices, we generate the uniform-purity ensemble by generating a uniform random number for the purity, and using it as a seed to generate a random probability vector on the spherical slice corresponding to that purity. In Fig. 1, the blue scatterplots refer to states generated from this uniform-purity ensemble of eigenvalue vectors. Once we generated the probability vector defining a random diagonal density matrix, the next and final step was to rotate it by a random unitary transformation to complete the random quantum state generation.

#### Step two: generating a random unitary transformation

Once we have both ensembles of randomly sampled diagonal density matrices, we rotate them by taking randomly selected unitary transformations whose distribution is uniform according to the Haar ensemble^33^. This is accomplished using the QR decomposition method employed in^34^ and discussed in^33^. Unitary matrices generated this way are uniformly distributed with respect to the Haar measure, and their uniformity is well-illustrated by the following point: if one produces a distribution of unitary matrices via this method, and then rotates each matrix in that distribution by the same (but arbitrary) unitary transformation, the distribution overall will remain unchanged. This invariance is similar to how a cluster of points uniformly distributed on the sphere remains uniformly distributed on the sphere however it is rotated.

### The data set of randomly generated density matrices

With the algorithm to generate random density matrices described, we generated two classes of ensembles of density matrices. The first ensemble has a uniformly sampled set of eigenvalues, while the second class of ensembles is uniform with respect to purity in order to generate more highly entangled states and better explore the effectiveness of different entanglement witnesses. In the table at the bottom of Fig. 1 we show that the percentages of randomly generated states whose entanglement is witnessed by mixedness measures $S_{\alpha }(A|B)$ increase when moving from the uniform to the uniform-purity ensembles and dramatically so at higher dimension. Indeed, the probability that a state selected from the uniform purity ensemble is “nearly pure” is bounded below by 1/2, while it decreases exponentially toward zero for the fully uniform ensemble.

In Fig. 1, we show scatter plots of the von Neumann conditional entropy $S_{1}(A|B)$ versus the second-order Renyi conditional entropy $S_{2}(A|B)$ obtained from the purity. When a state demonstrates entanglement by showing $S_{2}(A|B)<0$ , but not by $S_{1}(A|B)>0$, we say that the collision entropy has the advantage. In the alternate situation, we say the von Neumann entropy has the advantage. For two-qubit states, there do not appear to be any states for which the von Neumann entropy has the advantage. For the fully uniform and constant purity ensembles plotted here, it also appears that there are no states where the von Neumann entropy has the advantage, which implies that comparing purities will always be a more sensitive entanglement witness than comparing von Neumann entropies. However, this is not entirely the case.

Prior to developing a method of sampling uniformly at constant purity^34^, we had generated ensembles that covered a larger range of purities by taking the fully uniform ensemble, raising the (diagonal) density matrices to a given power based on the marginal dimension *D* of the $D\otimes D$ states, and renormalizing. This new distribution of density matrices was highly non-uniform at constant purity, but covered a larger range of purity values than the fully uniform ensemble to fill out the scatterplots. In these ensembles, we found for $3\otimes 3$ and $5\otimes 5$ states, that there do exist anomalous states for which the von Neumann entropy has the advantage. Upon examining these ensembles of $3\otimes 3$ and $5\otimes 5$ anomalous states, we found that they all have at least one thing in common. The joint and marginal density matrices for these states have approximately equal rank in that the *D* largest joint eigenvalues of the $D\otimes D$ system contain almost all of the total probability. With this restriction, the joint purity is approximately bounded by the same range of values as the marginal purity. As one can see in the in-set plots of the histograms of joint purity in Fig. 1, the likelihood of generating these anomalous states appears to be vanishingly small for the fully uniform ensemble.

#### Vanishing likelihood of anomalous states in uniform purity ensemble

As for why the uniform-*purity* ensemble also produces seemingly no anomalous states (among the samples we have generated), we can consider the likelihood of a high-dimensional state for a given value of purity also having a low rank. For a joint density matrix of dimension $N=D^{2}$, the fraction of joint states of rank no larger than *D* is essentially zero because such states would reside on facets or edges at the boundary of the probability simplex, amounting to an infinitesimal fraction of the total volume. Where the anomalous states generated have only approximately low rank (with otherwise many, but very small nonzero probabilities), the probability of generating states that are very close to these boundaries is still correspondingly small. This fact remains true, even when sampling uniformly at a constant purity, because a uniform sample of the $N-1$ dimensional probability simplex at constant purity is still an $N-2$ dimensional piecewise manifold. The additional constraint of the joint density matrix having rank no larger than *D* places the sample at a boundary of this piecewise manifold.

As a concrete example of these anomalous states, we can consider a mixture of three orthogonal, $3\otimes 3$ partially entangled states:

4 $$\begin{aligned} {\hat{\rho }}=p_{1}|\psi _{1}\rangle \langle \psi _{1}| + p_{2}|\psi _{2}\rangle \langle \psi _{2}| + p_{3}|\psi _{3}\rangle \langle \psi _{3}| \end{aligned}$$

such that

5a $$\begin{aligned} |\psi _{1}\rangle&= \sqrt{\lambda _{1}}|0,0\rangle + \sqrt{\lambda _{2}}|1,1\rangle + \sqrt{\lambda _{3}}|2,2\rangle \end{aligned}$$

5b $$\begin{aligned} |\psi _{2}\rangle&= \sqrt{\lambda _{1}}|1,0\rangle + \sqrt{\lambda _{2}}|2,1\rangle + \sqrt{\lambda _{3}}|0,2\rangle \end{aligned}$$

5c $$\begin{aligned} |\psi _{3}\rangle&= \sqrt{\lambda _{1}}|2,0\rangle + \sqrt{\lambda _{2}}|0,1\rangle + \sqrt{\lambda _{3}}|1,2\rangle \end{aligned}$$

Where $|\psi _{1}\rangle$, $|\psi _{2}\rangle$, and $|\psi _{3}\rangle$ are all mutually orthogonal, the joint entropy $S_{\alpha }(AB)$ is purely determined by the probability vector $(p_{1},p_{2},p_{3})$. Where the set of Schmidt coefficients associated to the measurement outcomes of system *B* is the same for $|\psi _{1}\rangle$, $|\psi _{2}\rangle$, and $|\psi _{3}\rangle$, the marginal entropy $S_{\alpha }(B)$ is determined purely by the probability vector $(\lambda _{1},\lambda _{2},\lambda _{3})$. Because we can choose $(p_{1},p_{2},p_{3})$ independently of $(\lambda _{1},\lambda _{2},\lambda _{3})$, it is straightforward to make an anomalous state where these two probability vectors are incomparable, and where the von Neumann entropy has the advantage at witnessing entanglement.

#### Relative effectiveness for different classes of states

To give an idea of how large the scatterplots in Fig. 1 might be with an exhaustive set of density matrices, we have used upper and lower bounds for von Neumann entropy for a constant collision entropy (i.e., constant purity) to enclose neighborhoods associated to broad classes of quantum states in Fig. 1. For both the set of pure states (small green blade), and states with maximally mixed marginals (large purple blade), the conditional entropies are expressed (up to a constant offset) as either marginal or joint entropies.

Where every point inside either blade and in the gap between them (explained momentarily) corresponds to a valid density matrix, we see that either ensemble of density matrices does not cover the full spectrum of values that these entropies can take, demonstrating their relative rarity. Even starting from a uniform distribution of pure states, the distribution of marginal eigenvalue spectra from these pure states is heavily weighted against high entanglement, as discussed in Appendix C of the Supplementary Material.

In the scatterplots in Fig. 1, the region enclosed between the two blades also corresponds to valid density matrices, and can be understood in the following way. The operation of mixing a pure state with a maximally mixed state is a continuous transformation of the density matrix, which must ultimately transform every pure state into one with a maximally mixed marginal, but which remains a valid quantum state for every value of mixing. Since the end points of the blade in the scatter plots are both pure states and ones with maximally mixed marginals, any curve connecting those two points that starts within the neighborhood must pass through every point in the gap between the two blades. Thus, there is a valid quantum state for every point in the gap between these two blades.

Although it can be clearly seen in the neighborhoods of Fig. 1, here we prove that for these classes of states, comparing purities will always witness entanglement before comparing von Neumann entropies. The family of Renyi entropies of order $\alpha$ is a decreasing function of $\alpha$. For the same density matrix, the collision entropy $S_{2}$ is less than or equal to the von Neumann entropy $S_{1}$. With these facts together, we can say the following.

Pure states are entangled if and only if their marginal subsystems are not also pure. All entangled pure states can be witnessed by comparing any measure of mixedness between joint and marginal states. Knowing this, we can understand that there is no joint pure state whose entanglement is witnessed by $S_{1}(A|B)<0$ that is not also witnessed by $S_{2}(A|B)<0$.

For joint states with maximally mixed marginals, the conditional entropy is equal to the joint entropy minus a constant offset. Since the joint collision entropy $S_{2}(AB)$ must be less than the joint von Neumann entropy $S_{1}(AB)$, it follows again that within this class of states, there also can be none where $S_{1}(A|B)<0$, but $S_{2}(A|B)>0$.

#### Increased sensitivity when using higher-order entropies

Using higher-order moments of the density matrix may yield more sensitive entanglement witnesses than the purity, but at the expense of becoming progressively more difficult to obtain from experiment. In particular, the direct measurement of $\text {Tr}[{\hat{\rho }}^{n}]$ requires interfering *n* copies of the state ${\hat{\rho }}$, which becomes intractable as *n* grows large. Indeed, determining the eigenvalue spectrum of a thirty-qubit quantum state would require interfering over $10^{9}$ copies of the state.

That said, it is straightforward to show that for all states with maximally mixed marginal systems, every state whose entanglement is witnessed by $S_{\alpha }(A|B)<0$ must have its entanglement witnessed with any entropy of higher order $\alpha '>\alpha$. This comes from the fact that the Renyi entropy of order $\alpha$ is a monotonically decreasing function of $\alpha$. From this we may conclude that for this class of states (which includes both Werner states and isotropic states) that comparing joint and marginal mixedness using higher order moments of the density matrix will be progressively more sensitive at witnessing entanglement than comparing von Neumann entropies or purities.

As a particularly striking example of how sensitive these higher-order entropies can be, we consider the case of the $N=D^{2}$-dimensional Werner state, which is a mixture of the Bell state $|\Phi \rangle \langle \Phi |$ and the maximally mixed state:

6a $$\begin{aligned} \rho _{AB}^{(Werner)}&=p |\Phi \rangle \langle \Phi | + (1-p)\frac{{\textbf{I}}}{D^2}, \end{aligned}$$

6b $$\begin{aligned} |\Phi \rangle&\equiv \frac{1}{\sqrt{D}}\sum _{i=1}^{D}|i\rangle |i\rangle . \end{aligned}$$

The probability eigenvalue vectors for the Werner state are:

7a $$\begin{aligned} \vec {\lambda }(AB)&=\Big (p+\frac{1-p}{D^{2}},\frac{1-p}{D^{2}},\ldots ,\frac{1-p}{D^{2}}\Big ), \end{aligned}$$

7b $$\begin{aligned} \vec {\lambda }(A)&=\vec {\lambda }(B)=\Big (\frac{1}{D},\ldots ,\frac{1}{D}\Big ). \end{aligned}$$

The entanglement of the Werner state is witnessed whenever $S_{\alpha }(A|B)<0$. For constant *p*, $S_{\alpha }(A|B)$ decreases as $\alpha$ increases; and for constant $\alpha$, $S_{\alpha }(A|B)$ decreases as *p* increases. To keep the value of $S_{\alpha }(A|B)$ constant at increasing $\alpha$, there must also be a corresponding decrease in *p*. The threshold Bell state fraction *p* for which $S_{\alpha }(A|B)=0$ must also decrease as $\alpha$ increases. See plots in Fig. 2 for example.

Clearly for Werner states, higher-order Renyi entropies make for more sensitive witnesses of entanglement than lower order. Indeed, if one uses $S_{1}(A|B)$, one finds that the threshold value of *p*, ($p_{c}$), does not scale favorably at high dimension. Instead, $p_{c}$ asymptotically approaches 1/2 as $N\rightarrow \infty$. On the other hand, using $S_{2}(A|B)$ scales more favorably, and has an analytic value of $p_{c}=1/\sqrt{D+1}$ (where $N=D^{2}$), decreasing toward zero for large dimension. Going beyond second order, using $S_{\infty }(A|B)$ scales better still, with an analytic value of $p_{c}=1/(D+1)$, a quadratic improvement over the collision entropy. Indeed, it was shown in Ref.^35^ that for $D\otimes D$ Werner states, $p_{c}=1/(D+1)$ is the necessary and sufficient critical value distinguishing separable states from entangled ones. Even here, the favorability of the scaling is understated. Recall that the 127-qubit state has dimension of $2^{127}\approx 1.7\times 10^{38}$, and a Werner state of such a dimension can still have its entanglement witnessed by comparing purities for any Bell state fraction greater than $7.67\times 10^{-20}$.

To examine the more general case of success in entanglement witnessing, we have used the data from the uniform purity ensembles for $3\otimes 3$ and $10\otimes 10$ systems, and plotted the different conditional entropy functions as well as the (logarithmic) negativity $E_{{\mathscr {N}}}({\hat{\rho }})$ as a function of joint state purity in Fig. 2. Although there is a substantial amount of noise at low dimension, we can clearly see as in the Werner state case, that the range of purities at which entanglement can be witnessed expands when using higher-order entropy.

### Case of unequal dimensions: enhanced entanglement detection with larger ancillae

Thus far, we have sampled joint states whose subsystems have equal dimension. It is worth considering whether comparing joint vs marginal purities maintains its advantage over von Neumann entropies in witnessing entanglement when the subsystem dimensions are unequal. To answer this question, we randomly generated $10^{6}$ joint diagonal density matrices of dimension $N=60$ according to the uniform-purity ensemble, performed random joint unitaries to obtain arbitrary density matrices, and subdivided them into $D_{A}\otimes D_{B}$ systems, where the dimension of subsystem *A*, $D_{A}$ is an element of the set $\{2,3,4,5,6,10,12,15,20,30\}$, and $D_{B}$ is its compliment such that $D_{A}\cdot D_{B}=N=60$. For each possible bipartition, a different random unitary was applied to the joint density matrix.

**Figure 3:**
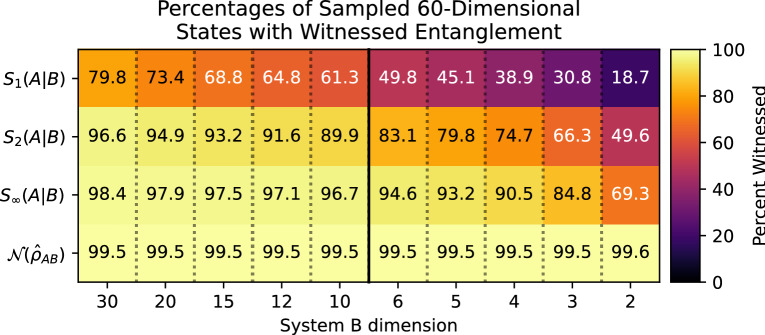
Plot showing percentages of 60-dimensional joint systems sampled according to the uniform-purity ensemble, whose entanglement was witnessed by comparing different forms of joint and marginal entropies, and by the negativity of the partial transpose. These systems were bipartitioned into subsystems of dimensions $D_{A}$ and $D_{B}$ respectively, such that $D_{A}D_{B}=60$. Starting with $10^{6}$ random diagonal density matrices, different random unitaries were performed for each possible bipartition to generate the full ensemble of density matrices analyzed here. The entanglement-success percentages are plotted as a function of $D_{B}$. The vertical axis denotes the different entanglement witnesses used, and in particular, that we are conditioning on subsystem *B*.

As shown in Fig. 3, we find that when the dimensions of the systems are unequal, comparing joint and marginal purities to witness entanglement still performs better than comparing joint and marginal von Neumann entropies. Beyond this, we also discover that when the dimensions of the subsystems are unequal, conditioning on the larger (higher-dimension) subsystem is more likely to witness entanglement than conditioning on the smaller one (all other factors constant). This distinction becomes most dramatic at the extreme case of $2\otimes 30$ systems, where (for say, comparing purities) conditioning on the qubit witnesses entanglement only 49.6% of the time, while conditioning on the quDit for $D=30$ witnesses entanglement at 96.6% of the time. Note that for the negativites, the sampling percentages are indistinguishable from being constant, up to the random sampling uncertainty of the subsystems.

## Discussion: correlations vs negativity in witnessing entanglement

In this work, we have shown that all methods of witnessing entanglement between two parties *A* and *B* by comparing the mixedness of the joint state to that of the marginals are subsumed by the negativity of the partial transpose. Given the close relationship between entanglement and correlation, we quickly discuss how many, but not all correlation-based witnesses are also subsumed by the negativity.

For two parties *A* and *B* sharing a pure quantum state between them $|\psi \rangle _{AB}$, all correlations are identifiable as entanglement, and the strength of those correlations corresponds to the amount of entanglement present. This relationship between correlation and entanglement is preserved for mixed states up to the amount of mixing present. For a pair of observables ${\hat{X}}_{A}$ and ${\hat{X}}_{B}$ of a joint quantum system with density operator ${\hat{\rho }}_{AB}$ whose correlations are quantified by the mutual information $H(X_{A}:X_{B})$, the relationship correlation and entanglement, relative to mixedness can be given by the relation:

8 $$\begin{aligned} H(X_{A}:X_{B}) \le E_{F}(A,B) + S(AB), \end{aligned}$$

where $E_{F}(A,B)$ is the entanglement of formation between systems *A* and *B* of ${\hat{\rho }}_{AB}$^36^, and *S*(*AB*) is the von Neumann entropy of ${\hat{\rho }}_{AB}$. See Appendix E of the Supplementary Material for proof. This relation shows that beyond the mixedness of the joint state, there can be no correlations without entanglement. Importantly, this means that for nearly pure states with joint quantum entropy *S*(*AB*) near zero, nearly all correlations can be identified as entanglement. However, this relation is based on negative values of the quantum conditional entropy *S*(*A*|*B*) being lower limits to entanglement measures such as the entanglement of formation $E_{F}$. Because of this, many classes of correlation-based entanglement witnesses including many EPR-steering inequalities^37–40^ are subsumed by the negativity of the partial transpose, but not all of them.

In Ref.^41^, there are Bell inequalities that can witness the entanglement in bound-entangled states with a positive partial transpose. Where Bell inequalities fundamentally identify entanglement through correlations, we see that the relationship between entanglement and correlations is more subtle than relative mixedness can describe.

## Conclusion: merits of different entanglement witnesses

In our investigations, we examined how well comparing the mixedness of a joint quantum state to the mixedness of its subsystems witnesses entanglement. We illustrated how entanglement is witnessed this way, and proved that states whose entanglement can be witnessed this way must have a negative partial transpose (NPT). In this way, we understand that the negativity of the partial transpose supersedes all entanglement-witnessing strategies based on showing that the joint system is less mixed than its marginals.

Because it is not always practical to determine the negativity of a high dimensional state, we also examined how well different popular measures of mixedness witness entanglement on large ensembles of systems of varying dimension. Our Monte Carlo simulations revealed that comparing joint and marginal purity is overwhelmingly (though not exclusively) more successful than comparing joint and marginal entropies even though it requires fewer resources. This is promising, as there exist direct measurements of quantum state purity by interfering two copies of a quantum system^25,42^, so that full state tomography is unnecessary.

## Supplementary Information

Supplementary Information.

## Data Availability

The datasets used and/or analysed to generate the plots and related statistics in the body of this article are available from the corresponding author upon reasonable request.

## References

[1] Feynman RP (1986). Quantum mechanical computers. Found. Phys..

[2] Knill, E., Laflamme, R. & Milburn, G. J. A scheme for efficient quantum computation with linear optics. *Nature* **409**, 46–52 (2001). 10.1038/35051009 11343107

[3] Bharti K (2022). Noisy intermediate-scale quantum algorithms. Rev. Mod. Phys..

[4] Bennett CH (1993). Teleporting an unknown quantum state via dual classical and einstein-podolsky-rosen channels. Phys. Rev. Lett..

[5] Ekert AK (1991). Quantum cryptography based on bell’s theorem. Phys. Rev. Lett..

[6] Deng F-G, Long GL, Liu X-S (2003). Two-step quantum direct communication protocol using the einstein-podolsky-rosen pair block. Phys. Rev. A.

[7] Zhang W (2017). Quantum secure direct communication with quantum memory. Phys. Rev. Lett..

[8] Qi, Z. *et al.* A 15-user quantum secure direct communication network. *Light Sci. Appl.* **10**, 1–8 (2021). 10.1038/s41377-021-00634-2 PMC8440625 34521809

[9] Sheng Y-B, Zhou L, Long G-L (2022). One-step quantum secure direct communication. Sci. Bull..

[10] Giovannetti V, Lloyd S, Maccone L (2004). Quantum-enhanced measurements: Beating the standard quantum limit. Science.

[11] Giovannetti V, Lloyd S, Maccone L (2006). Quantum metrology. Phys. Rev. Lett..

[12] Lloyd, S. Enhanced sensitivity of photodetection via quantum illumination. *Science* **321**, 1463–1465. 10.1126/science.1160627 (2008). 10.1126/science.1160627 18787162

[13] Chow, J., Dial, O. & Gambetta, J. Ibm quantum breaks the 100-qubit processor barrier. *IBM Res. Blog*, available in https://research.ibm.com/blog/127-qubit-quantum-process-or-eagle (2021).

[14] Zhong H-S (2020). Quantum computational advantage using photons. Science.

[15] Schneeloch J, Tison CC, Fanto ML, Alsing PM, Howland GA (2019). Quantifying entanglement in a 68-billion-dimensional quantum state space. Nat. Commun..

[16] Peres A (1996). Separability criterion for density matrices. Phys. Rev. Lett..

[17] Roberts, A. W. & Varberg, D. E. *Convex functions*, 258–259 (Academic Press, 111 Fifth Avenue, New York, New York 10003, 1973).

[18] Nielsen MA, Kempe J (2001). Separable states are more disordered globally than locally. Phys. Rev. Lett..

[19] Horodecki M, Horodecki P, Horodecki R (1996). Separability of mixed states: Necessary and sufficient conditions. Phys. Lett. A.

[20] Horodecki M, Horodecki P, Horodecki R (1998). Mixed-state entanglement and distillation: Is there a “bound” entanglement in nature?. Phys. Rev. Lett..

[21] Horodecki, P., Rudnicki, Ł. & Życzkowski, K. Five open problems in quantum information. arXiv:2002.03233 (2020).

[22] Hiroshima T (2003). Majorization criterion for distillability of a bipartite quantum state. Phys. Rev. Lett..

[23] Gühne O, Tóth G (2009). Entanglement detection. Phys. Rep..

[24] Bourennane M (2004). Experimental detection of multipartite entanglement using witness operators. Phys. Rev. Lett..

[25] Ekert AK (2002). Direct estimations of linear and nonlinear functionals of a quantum state. Phys. Rev. Lett..

[26] Bruni TA (2004). Measurimg polynomial functions of states. Quant. Inf. Comput..

[27] Brydges T (2019). Probing rényi entanglement entropy via randomized measurements. Science.

[28] Yu X-D, Imai S, Gühne O (2021). Optimal entanglement certification from moments of the partial transpose. Phys. Rev. Lett..

[29] Elben A (2020). Mixed-state entanglement from local randomized measurements. Phys. Rev. Lett..

[30] Zhou Y, Zeng P, Liu Z (2020). Single-copies estimation of entanglement negativity. Phys. Rev. Lett..

[31] Gray J, Banchi L, Bayat A, Bose S (2018). Machine-learning-assisted many-body entanglement measurement. Phys. Rev. Lett..

[32] Życzkowski K, Horodecki P, Sanpera A, Lewenstein M (1998). Volume of the set of separable states. Phys. Rev. A.

[33] Mezzadri F (2007). How to generate random matrices from the classical compact groups. Not. AMS.

[34] Alsing PM, Tison CC, Schneeloch J, Birrittella RJ, Fanto ML (1999). Distribution of density matrices at fixed purity for arbitrary dimensions. Phys. rev. res..

[35] Horodecki M, Horodecki P (1999). Reduction criterion of separability and limits for a class of distillation protocols. Phys. Rev. A.

[36] Wootters WK (1998). Entanglement of formation of an arbitrary state of two qubits. Phys. Rev. Lett..

[37] Berta M, Christandl M, Colbeck R, Renes JM, Renner R (2010). The uncertainty principle in the presence of quantum memory. Nat. Phys..

[38] Walborn SP, Salles A, Gomes RM, Toscano F, Souto Ribeiro PH (2011). Revealing hidden Einstein–Podolsky–Rosen nonlocality. Phys. Rev. Lett..

[39] Schneeloch J, Broadbent CJ, Walborn SP, Cavalcanti EG, Howell JC (2013). Einstein–Podolsky–Rosen steering inequalities from entropic uncertainty relations. Phys. Rev. A.

[40] Schneeloch J, Howland GA (2018). Quantifying high-dimensional entanglement with Einstein–Podolsky–Rosen correlations. Phys. Rev. A.

[41] Vértesi T, Brunner N (2014). Disproving the Peres conjecture by showing bell nonlocality from bound entanglement. Nat. Commun..

[42] Bovino FA (2005). Direct measurement of nonlinear properties of bipartite quantum states. Phys. Rev. Lett..

