# Supplementary material for: Negativity vs. purity and entropy in witnessing entanglement
Source: Sci Rep. 2023 Mar 21;13:4601. doi: 10.1038/s41598-023-31273-9 (PMC10030834; doi:10.1038/s41598-023-31273-9)
Supplement: Supplementary file 1 — Supplementary Information. [file 41598_2023_31273_MOESM1_ESM.pdf]

Supplementary Material: Negativity vs. Purity and Entropy in Witnessing Entanglement

James Schneeloch[1,*], Christopher C. Tison[1], H Shelton Jacinto[1], and Paul M. Alsing[1]

[1]Air Force Research Laboratory, Information Directorate, Rome, New York, 13441, USA
[*]james.schneeloch.1@afrl.af.mil

ABSTRACT

In these appendices, we discuss in detail supplementary material for our article, “Negativity vs. Purity and Entropy in Witnessing Entanglement". In Appendix A, we lay out how the law of large numbers biases a naïve sampling of probability vectors toward maximally mixed distributions. In Appendix B, we discuss how with a uniform sampling, the probability of “nearly pure" and “nearly maximally mixed" states decreases rapidly at high dimension. In Appendix C, we discuss how a uniform sampling of pure states produces increasingly entangled states with increasing rarity, In Appendix D, we discuss how to obtain upper and lower bounds to von Neumann entropy for a given state purity, used to create the neighborhoods of states in Figure 1 of our article. In Appendix E, we provide a short proof for our entanglement-correlation relation discussed in Section 5 of our article.

Appendix A: The Law of Large Numbers and naïve sampling of probability vectors

In Section 4.1.1 of our article, we argue that a naïve sampling of a probability vector $\vec{p}$ of dimension N in which N random numbers are drawn from a probability distribution on the real numbers between zero and unity produces vectors overwhelmingly weighted toward the maximally mixed state. In particular, we can prove that where $H(\vec{p})$ is the Shannon entropy of a probability vector $\vec{p}$ obtained from this naïve sampling process, that:

$$H(\vec{p}) \rightarrow \log(N) + const, \tag{1}$$

where $\log(N)$ is the entropy of a maximally mixed probability vector of dimension N.

The law of large numbers (in particular, Borel's law of large numbers[1]) states that as the length of a sequence of independent and identically distributed random variables grows, the relative frequencies of each outcome of the random variable converge to their respective probabilities. If the probability of outcome j of random variable X is p, then for a sufficiently large number of trials, the fraction of trials for which the outcome of X was j will also converge to p.

Consider generating a probability vector $\vec{p}$ by the naïve sampling described at the beginning of this section. In each trial, we generate N random values according to the same fixed probability distribution for each value, and normalize them to randomly generate the N components of $\vec{p}$. Let the function $f(k)$ where k goes from 1 to N define the kth smallest generated probability component in $\vec{p}$ from this random sample. Next, we define the rescaled function $\tilde{f}(x) = N \times f(Nx)$ so that x goes from $1/N$ to one as k goes from one to N.

As the dimension N grows, the law of large numbers proves that $\tilde{f}(x)$ converges to some fixed probability density on the interval $[0,1]$, so that $f(k)$ forms a scaled version of $\tilde{f}(x)$ by factor N. This implies that the entropy of the naïvely generated probability vector $\vec{p}$ approaches $\log(N)$ plus a constant equal to the continuous entropy of $\tilde{f}(x)$:

$$H(\vec{p}) \rightarrow \log(N) + h(\tilde{f}(x)). \tag{2}$$

Here, we point out that $h(\tilde{f}(x))$ is the continuous Shannon entropy of probability density $\tilde{f}(x)$[2]; it is independent of dimension N, and becomes insignificant in the limit of large N. Where $\log(N)$ is the entropy of a maximally mixed probability vector of dimension N, it follows that probability vectors generated in this naïve fashion will be substantially biased toward maximally mixed distributions.

Appendix B: The probability of “Nearly pure” and “Nearly maximally mixed” states

In Section 4.1.1 of our article, we introduce the concept of a “nearly pure” probability vector as one in which the maximum component is greater than or equal to $1/2$. Geometrically, we can look at a vertex of the probability simplex, and select the halfway points on all lines connecting this vertex to other vertices. The convex hull of the vertex and this set of midway points is a simplex identical in shape to the probability simplex, but scaled down by a factor of 1/2 in every dimension. Within this

sub-simplex there are only nearly pure states because its proximity to the vertex of the probability simplex requires that the maximum probability component of any point in this sub simplex be at least $1/2$.

The ratio of the volume of the sub-simplex to the total probability simplex is $(1/2^{N-1})$ where N is the total dimension of the probability space (and the number of outcomes of the probability distribution). Moreover, there are only N of these sub-simplices containing the nearly pure states because there are only N vertices of the probability simplex. Putting these two facts together, the fraction of states on the probability simplex that are nearly pure $P_{\approx P}$ is:

$$P_{\approx P} = \frac{N}{2^{N-1}}. \tag{3}$$

For three dimensions (see Fig. 4), this fraction is $3/4$, but it decreases exponentially with dimension N.

To find the probability of the nearly maximally mixed states, we point out that the volume V_N of the N-dimensional probability simplex is:

$$V_N^{(simplex)} = \frac{\sqrt{N}}{(N-1)!}. \tag{4}$$

The set of nearly maximally mixed states is the set of states whose purity $\mathscr{P}$ is between $1/N$ and $1/(N-1)$. The volume in the simplex that these states occupy is of a uniform N-dimensional sphere, centered on the maximally mixed state, and with a radius $r = \sqrt{\frac{1}{N(N-1)}}$:

$$V_N^{(sphere)} = \frac{\pi^{\frac{N}{2}}}{\Gamma(\frac{N}{2}+1)} (r)^N. \tag{5}$$

The ratio of these two volumes gives the probability of a nearly maximally mixed state $P_{\approx MM}$ under uniform sampling to be:

$$P_{\approx MM} = \frac{1}{\sqrt{N}} \left(\frac{\pi}{N(N-1)}\right)^{N/2} \frac{\Gamma(N)}{\Gamma(N/2+1)}. \tag{6}$$

Where $\Gamma(N) \leq (N/2+1)^{(N/2-1)}\Gamma(N/2+1)$, and where in general $\Gamma(N/2+1) \ll \Gamma(N)$, we have:

$$P_{\approx MM} \ll \frac{1}{(N/2)\sqrt{N}} \left(\frac{\pi}{N}\right)^{N/2}. \tag{7}$$

In short, we see that the probability of nearly maximally mixed states decreases even faster than exponentially under uniform damping.

Appendix C: Rarity of highly entangled states even when uniformly sampling pure states

The algorithm for generating a uniform distribution of density matrices where: one takes a random eigenvalue vector uniform on the probability simplex, and then transforms the diagonal matrix with a random unitary matrix uniform on the Haar measure, is well-justified. However, when one interprets this uniform distribution of density matrices as a uniform distribution of $N = D \otimes D$ joint density matrices, the resulting distribution of marginal density matrices is far from uniform.

As an example, we consider the uniform distribution of $D \otimes D$ pure states generated from Haar-random unitaries acting on a single pure state. In[3], the distribution of marginal eigenvalues for this distribution of states is actually derived explicitly. The distribution of the eigenvalues of the D-dimensional subsystem taken from the partial trace of uniformly sampled $D \otimes D$-dimensional pure states is given by:

$$\rho(\vec{\lambda}, D) = \frac{\Gamma(D^2)}{\prod_{j=0}^{D-1}(\Gamma(D-j)\Gamma(D+1-j))} \cdot \delta(1 - \sum_i \lambda_i) \prod_{i<j}^{D} (\lambda_i - \lambda_j)^2. \tag{8}$$

where $\Gamma(x)$ is Euler's gamma function, and $\delta(x)$ is the Dirac delta function, employed here to enforce the constraint that the eigenvalues sum to unity.

In particular, we note that anywhere in the (marginal) probability simplex where two or more eigenvalues approach equality, the probability of generating such an eigenvalue vector approaches zero. See Fig. 1 for $3 \otimes 3$ example. Where the maximally entangled states occur as the marginal eigenvalues approach $1/D$ (equaling each other), the probability of generating highly entangled states becomes vanishingly small even when accounting for the fraction of the volume of the simplex where such states would reside.

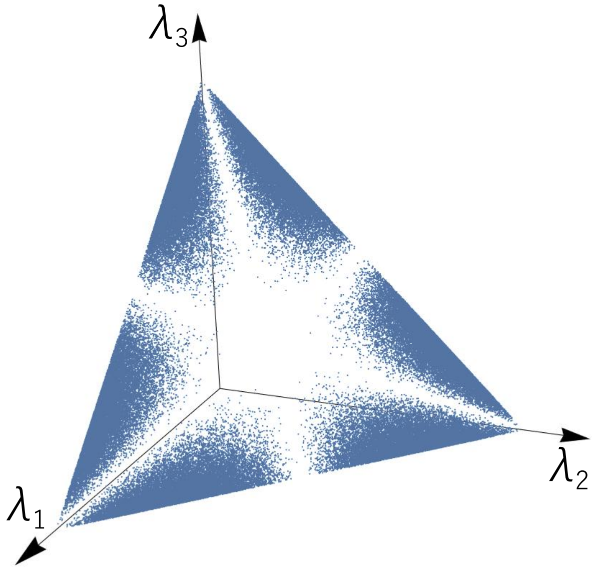

Figure 1. Scatterplot of marginal eigenvalue vectors $\vec{\lambda} = (\lambda_1, \lambda_2, \lambda_3)$ of the partial trace of uniformly sampled joint $3 \otimes 3$ pure states.

Appendix D: Upper and Lower bounds to von Neumann entropy given constant state purity.

While comparing joint and marginal purities seems to be more effective at witnessing entanglement than comparing von Neumann entropies, it is von Neumann entropies that have a larger utility in various applications in quantum information science. To this end, it is useful to point out straightforward upper and lower bounds to the von Neumann entropy for a given state purity.

In[4], Berry and Sanders provide these bounds, which we now discuss here. The maximum entropy distribution (given in equation (9) as $\vec{p}_{max}$) for constant purity $\mathscr{P}$ is uniform except for one outcome. In the probability simplex of N outcomes (see Fig. 4 for example), points on line segments going from the maximally mixed state to a vertex are the only distributions of this class, being equal in all coordinates except for one (not counting the maximally mixed state). Where the manifold of constant purity probability vectors forms an $N-2$ dimensional sphere centered on the maximally mixed state, the intersection of this manifold with these line segments gives the probability distributions of maximum entropy:

$$\vec{p}_{max} = (p_0, ..., p_0, 1-(N-1)p_0). \tag{9}$$

Given the form of $\vec{p}_{max}$, the purity $\mathscr{P}$ is readily expressed in terms of p_0:

$$\mathscr{P} = (N-1)p_0^2 + (1-(N-1)p_0)^2. \tag{10}$$

Of the two values of p_0 satisfying this constraint, the value of p_0 less than $1-(N-1)p_0$ is the value producing the maximum entropy distribution.

The *minimum* entropy probability distribution (given in equation (11)) for constant purity $\mathscr{P}$ is a discrete top-hat distribution appended with a final nonzero probability and all other probabilities zero. If we define κ as $1/\mathscr{P}$ rounded down to the nearest integer (see D.1 for discussion), then the minimum entropy probability distribution will have κ outcomes of equal nonzero probability, one outcome with lesser probability, and all other outcomes of probability zero:

$$\vec{p}_{min} = (p_0, ..., p_0, 1-\kappa p_0, 0, 0, ..., 0). \tag{11}$$

Except for the case of three dimensions ($N=3$), $\vec{p}_{min}$ and $\vec{p}_{max}$ do not both fall on the same lines passing through the maximally mixed state (See Fig. 4 for a diagram of the 3-dimensional case). In general, there will be more distributions of minimum von Neumann entropy for a given state purity than maximum entropy distributions because all permutations of $\vec{p}$ have the same entropy, and there are never fewer permutations of $\vec{p}_{min}$ than of $\vec{p}_{max}$.

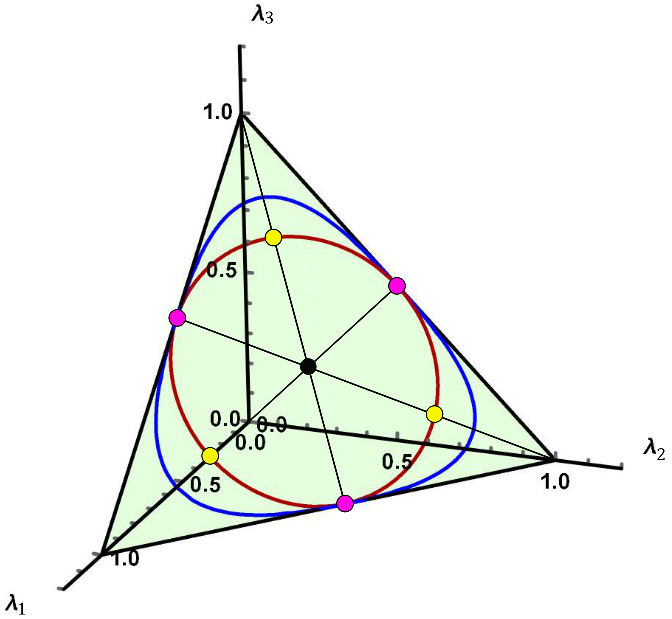

Figure 2. Plot of probability simplex of three outcomes ($\lambda_1 + \lambda_2 + \lambda_3 = 1$) with contours of constant purity (red circle) and constant von Neumann entropy (blue triangular loop), where $S_1 = S_2 = 1$ bit. As purity decreases and entropy increases, the contours shrink, converging to the maximally mixed state at the centroid of the simplex (black dot). For a constant purity, the maximum entropy distributions will lie at the intersections between the purity contour and the medial line segments going from the centroid to the vertices of the simplex (yellow circles). For this dimension, the minimum entropy distributions lie at the opposite intersections (magenta circles).

D.1: Note on the value κ

In[4], Berry and Sanders give the value κ in equation (11) as the value $1/p_0$ rounded down to the nearest integer instead of $1/\mathscr{P}$ rounded down to the nearest integer. Here, we show that both these expressions are equivalent.

Using the Berry-Sanders definition for κ, it follows that

$$\frac{1}{p_0} - 1 \leq \kappa \leq \frac{1}{p_0}. \tag{12}$$

Next, we know that $\vec{p}_{min}$ has between κ and $\kappa + 1$ outcomes, so it should be straightforward to prove that the purity $\mathscr{P}$ is bounded by:

$$\frac{1}{\kappa} \geq \mathscr{P} \geq \frac{1}{\kappa + 1}. \tag{13}$$

To prove this, we note that p_0 is also bounded between $1/(\kappa+1)$ and $1/\kappa$, and that within this range the purity $\mathscr{P}$ increases monotonically with p_0:

$$\mathscr{P} = \kappa p_0^2 + (1 - \kappa p_0)^2, \tag{14a}$$

$$\frac{\partial \mathscr{P}}{\partial p_0} = 2\kappa(p_0(\kappa+1) - 1), \tag{14b}$$

$$\frac{\partial \mathscr{P}}{\partial p_0} \geq 0 \qquad \text{where} \qquad p_0 \geq \frac{1}{\kappa+1}. \tag{14c}$$

From this, we can say that where $1/(\kappa+1)$ and $1/\kappa$ are valid values for the purity $\mathscr{P}$, $\mathscr{P}$ must increase monotonically as p_0 does from $1/(\kappa+1)$ to $1/\kappa$, proving equation (13).

Next, we can algebraically rearrange the inequalities in equation (13) into the relation:

$$\frac{1}{\mathscr{P}} - 1 \leq \kappa \leq \frac{1}{\mathscr{P}}. \tag{15}$$

As κ is defined to be an integer, we can conclude that while κ was originally defined as $1/p_0$ rounded down to the nearest integer, it is equivalent to the value of $1/\mathscr{P}$, rounded down to the nearest integer.

Appendix E: Proof of entanglement-correlation relation

In Section 5 of our article, we illustrated the relationship between correlation and entanglement through the relation:

$$H(X_A : X_B) \leq E_F(A,B) + S(AB), \tag{16}$$

This relationship is straightforward to show because the classical correlations between two parties A and B is bounded from above by the marginal quantum entropy demonstrated in[5]:

$$H(X_A : X_B) \leq \min\{S(A), S(B)\}, \tag{17}$$

To prove this, we follow the basic logic laid out in[5]. First, we note that the classical mutual information $H(X_A : X_B)$ is the quantum mutual information of AB after observables $\hat{X}_A$ and $\hat{X}_B$ have been measured. Because quantum mutual information is a form of relative entropy, we can use the monotonicity of relative entropy to say that the quantum mutual information decreases upon measurement, and in particular, that:

$$H(X_A : X_B) \leq H(X_A : B), \tag{18}$$

where the right-hand side is the quantum mutual information after only $\hat{X}_A$ has been measured.

After $\hat{X}_A$ has been measured, the joint state $\hat{\rho}_{AB}$ has a form known as classical-quantum:

$$\hat{\rho}_{X_A B} = \sum_i P(X_{Ai}) |X_{Ai}\rangle\langle X_{Ai}| \otimes \hat{\rho}_{Bi}. \tag{19}$$

The mutual information of this kind of state[6]: is given by:

$$H(X_A : B) = S(B) - \sum_i P(X_{Ai}) S(\hat{\rho}_{Bi}). \tag{20}$$

Since von Neumann entropy (without conditioning) is non negative, we obtain the bound:

$$H(X_A : B) \leq S(B). \tag{21}$$

Together, this gives us the relation:

$$H(X_A : X_B) \leq S(B). \tag{22}$$

Since we can perform the same set of steps with system B being measured instead, we see that $H(X_A : X_B)$ cannot exceed either $S(A)$ or $S(B)$, thus proving the correlation relation (17) demonstrated in[5].

Next, we express $\min\{S(A), S(B)\}$ in terms of conditional and joint entropy:

$$\min\{S(A), S(B)\} = \min\left\{ \begin{matrix} S(AB) - S(B|A) \\ , S(AB) - S(A|B) \end{matrix} \right\} = \min\{-S(B|A), -S(A|B)\} + S(AB). \tag{23}$$

Where the quantum conditional entropy is concave, we have for any pure state decomposition:

$$\begin{aligned} \min\{S(A), S(B)\} &\leq S(AB) + \min\left\{ \sum_i p_i(-S_i(B|A)), \sum_i p_i(-S_i(A|B)) \right\}, \\ &= \min\left\{ \sum_i p_i S_i(A), \sum_i p_i S_i(B) \right\} + S(AB), \\ &= \min\left\{ \sum_i p_i S_i(A) \right\} + S(AB). \end{aligned} \tag{24}$$

Where the same pure state decomposition is taken for both sums, we must have that $S_i(A) = S_i(B)$. Since this relation must be true for all pure state decompositions, we can choose the minimizing pure-state decomposition, which defines the entanglement of formation:

$$E_F(A,B) \equiv \min_{|\psi\rangle} \left\{ \sum_i p_i S_i(A) \right\}. \tag{25}$$

Combining this with the previous inequality gives us the relation:

$$\min\{S(A),S(B)\} \leq E_F(A,B)+S(AB). \tag{26}$$

Incorporating this into the correlation relation (17) gives us the entanglement-correlation relation:

$$H(X_A:X_B) \leq E_F(A,B)+S(AB). \tag{27}$$

Important note: Because this relation (27) exists at all finite dimension with neither side explicitly dependent on dimension, it exists in the continuum limit [See[7] for example of use of continuum limit]. Where the mutual information between a pair of continuous observables is given by $h(x_A:x_B)$, the corresponding entanglement-correlation relation is given by:

$$h(x_A:x_B) \leq E_F(A,B)+S(AB). \tag{28}$$

References

1. Tomkins, R. J. Another proof of borel's strong law of large numbers. *The Am. Stat.* **38**, 208–209, DOI: 10.1080/00031305.1984.10483203 (1984).
2. Cover, T. M. & Thomas, J. A. *Elements of Information Theory* (Wiley and Sons, New York, 2006), second edn.
3. Bengtsson, I. & Życzkowski, K. *Geometry of Quantum States: An Introduction to Quantum Entanglement*, chap. 15, 658–661 (Cambridge University Press, 2017), second edn.
4. Berry, D. W. & Sanders, B. C. Bounds on general entropy measures. *J. Phys. A: Math. Gen.* **36**, 12255 (2003).
5. Hall, M. J. W., Andersson, E. & Brougham, T. Maximum observable correlation for a bipartite quantum system. *Phys. Rev. A* **74**, 062308, DOI: 10.1103/PhysRevA.74.062308 (2006).
6. Wilde, M. M. *Quantum information theory* (Cambridge University Press, 2017), second edn.
7. Schneeloch, J. & Howland, G. A. Quantifying high-dimensional entanglement with einstein-podolsky-rosen correlations. *Phys. Rev. A* **97**, 042338, DOI: 10.1103/PhysRevA.97.042338 (2018).
